# Comparative transcriptomic analysis of candidate effectors to explore the infection and survival strategy of *Bursaphelenchus xylophilus* during different interaction stages with pine trees

**DOI:** 10.1186/s12870-021-02993-9

**Published:** 2021-05-19

**Authors:** Long-Jiao Hu, Xiao-Qin Wu, Xiao-Lei Ding, Jian-Ren Ye

**Affiliations:** 1 Co-Innovation Center for Sustainable Forestry in Southern China, College of Forestry, Nanjing Forestry University, Nanjing, 210037 China; 2 Jiangsu Key Laboratory for Prevention and Management of Invasive Species, Nanjing Forestry University, Nanjing, 210037 China

**Keywords:** *Bursaphelenchus xylophilus*, RNA-Seq, Comparative transcriptome, Candidate effectors, Cell death suppression

## Abstract

**Background:**

The pine wood nematode (PWN), *Bursaphelenchus xylophilus*, is a devastating pathogen of many *Pinus* species in China. The aim of this study was to understand the interactive molecular mechanism of PWN and its host by comparing differentially expressed genes and candidate effectors from three transcriptomes of *B. xylophilus* at different infection stages.

**Results:**

In total, 62, 69 and 46 candidate effectors were identified in three transcriptomes (2.5 h postinfection, 6, 12 and 24 h postinoculation and 6 and 15 d postinfection, respectively). In addition to uncharacterized pioneers, other candidate effectors were involved in the degradation of host tissues, suppression of host defenses, targeting plant signaling pathways, feeding and detoxification, which helped *B. xylophilus* survive successfully in the host. Seven candidate effectors were identified in both our study and the *B. xylophilu*s transcriptome at 2.5 h postinfection, and one candidate effector was identified in all three transcriptomes. These common candidate effectors were upregulated at infection stages, and one of them suppressed pathogen-associated molecular pattern (PAMP) PsXEG1-triggered cell death in *Nicotiana benthamiana.*

**Conclusions:**

The results indicated that *B. xylophilus* secreted various candidate effectors, and some of them continued to function throughout all infection stages. These various candidate effectors were important to *B. xylophilus* infection and survival, and they functioned in different ways (such as breaking down host cell walls, suppressing host defenses, promoting feeding efficiency, promoting detoxification and playing virulence functions). The present results provide valuable resources for in-depth research on the pathogenesis of *B. xylophilus* from the perspective of effectors.

**Supplementary Information:**

The online version contains supplementary material available at 10.1186/s12870-021-02993-9.

## Background

As one of the most serious coniferous forest pathogens, the pine wood nematode (PWN), *Bursaphelenchus xylophilus*, is an essential threat to forest ecosystems worldwide, and pine wilt disease (PWD) caused by *B. xylophilus* has resulted in massive economic losses in Asian and European countries, especially China and Japan [1, 2]. In China, the occurrence of PWD has been distributed in 18 provinces (No. 4 bulletin in 2020, National Forestry and Grassland Administration, China).

*B. xylophilus* is a migratory phytoparasitic nematode that has a unique feeding strategy, which includes phytophagous and mycetophagous stages, enabling it to reproduce and survive in host pines. Due to the complexity of the interaction between *B. xylophilus* and its host, the mechanisms of pathogenesis remain unclear.

Effectors are key elements in the virulence of various pathogens and parasites (including fungi, oomycetes, bacteria and plant-parasitic nematodes [PPNs]) against plants [3–6]. The effectors of PPNs are secreted into host plant tissues and facilitate invasion and migration to modulate the host immune system [7]. To determine the functions of effectors of PPNs in the interaction process between PPNs and their hosts, it is essential to screen and identify effectors.

Compared to microarrays and expressed sequence tags (ESTs), RNA-seq allows simultaneous transcript discovery and abundance estimation as well as identification of associated molecular cellular pathways and effectors of pathogens secreted during the infection process [8]. Many pathogen effectors have been identified by this reliable method. For example, putative effector proteins of the *Gymnosporangium yamadae* and *G. asiaticumrice* rust species, the *Heterodera avenae* cereal cyst nematode, the *Hirschmanniella oryzae* root nematode and the *Pratylenchus penetrans* root lesion nematode that may alter host defense mechanisms have been screened by RNA-seq [9–12]. At present, transcriptome sequencing has also been used to identify differentially expressed genes (DEGs) when *B. xylophilus* enters the initial phytophagous phase (2.5 h postinoculation) [13]. Espada et al. predicted candidate *B. xylophilus* effectors by comparing transcriptomes between the mycetophagous and middle and later phytophagous parasitic stages (6 and 15 d postinoculation) [14]. Moreover, in our recent study, we successfully identified and characterized three effectors (BxSapB1, BxSapB3 and Bx-FAR-1) and a novel molecular pattern, BxCDP1, from the transcriptomes of *B. xylophilus* during the mycetophagous and earlier infection stages (6, 12 and 24 h postinoculation) [15–18]. However, our transcriptome data were only used to screen candidate effector lists, and a large amount of data has not been thoroughly analyzed, including the types of upregulated genes, the functional annotation, the molecular cellular pathways of the involved candidate effectors and the internal relationships between candidate effectors. Moreover, previous studies have shown that the effector pathogens secreted are different in the different infection stages [10, 19].

In this study, our objective was to determine the roles of candidate effectors in *B. xylophilus* infection and survival by comparing the types of candidate effectors secreted by *B. xylophilus* during interactions with host trees at different infection stages. For this purpose, we performed a relative comparative transcriptomic analysis of *B. xylophilus* inoculated onto pines between our data and the above two previously reported *B. xylophilus* transcriptomic datasets from different stages of infection (2.5 h, 6 and 15 d postinfection). Particular emphasis was placed on the identification of DEGs, comparison of DEGs, Gene Ontology (GO) enrichment analysis and Kyoto Encyclopedia of Genes and Genomes (KEGG) pathway analysis of candidate effectors of *B. xylophilus* from the three transcriptomic groups. The relative expression of five common candidate effectors from three *B. xylophilu*s transcriptomes was detected at infection stages by quantitative real-time polymerase chain reaction (qRT-PCR). Moreover, the transient expression of the five common candidate effectors was characterized via a potato virus X (PVX) expression vector in *Nicotiana benthamiana*.

## Results

### De novo assembly of the transcriptome and comparison of summary data from three transcriptomes for *Bursaphelenchus xylophilus*

In total, we generated 592,991,982 raw reads and 570,138,674 clean reads from 12 samples by Illumina sequencing, respectively, which have been shown in our previous study [15]. The Pearson correlation between these samples was calculated (Fig. S1). In this study, we denoted our transcriptome sequencing data and two previously reported *B. xylophilus* transcriptomic datasets from different stages of infection at 2.5 h, 6 and 15 d postinfection as the B group, A group and C group, respectively. The comparison of summary data from the three transcriptomes (A, B and C group) for *B. xylophilus* is shown in Table S1. The comparative results showed that the numbers of raw reads and clean reads of samples from the A group and C group were much lower than those from our experimental data (B group).

### Screening of upregulated genes during the infection stage of *B. xylophilus*

In our study, the genes with a *P*-value < 0.05 and log_2_ (fold change) > 1 were assigned as DEGs between the early stages of infection (6, 12 and 24 h postinfection) and the mycetophagous stage (0 h). Finally, a total of 867 DEGs were obtained. Of these DEGs, 247 were upregulated during at least one of the phytophagous time points compared with the mycetophagous stage. A Venn diagram and heatmap of DEGs at the mycetophagous stage and three early phytophagous parasitic stages are shown in Figs. S2-S3. Of the 2272 DEGs in the A group, 1143 genes were upregulated in at least one postinoculation sample. Compared to the control, 60 genes were upregulated in both postinoculation samples [13]. In the previous literature, there was no specific indication of the number of different genes in the C group, but the top 200 sequences upregulated in the parasitic life stage of the nematode were identified [14].

### Functional annotation of upregulated genes from *B. xylophilus* transcriptomes

**Fig. 1:**
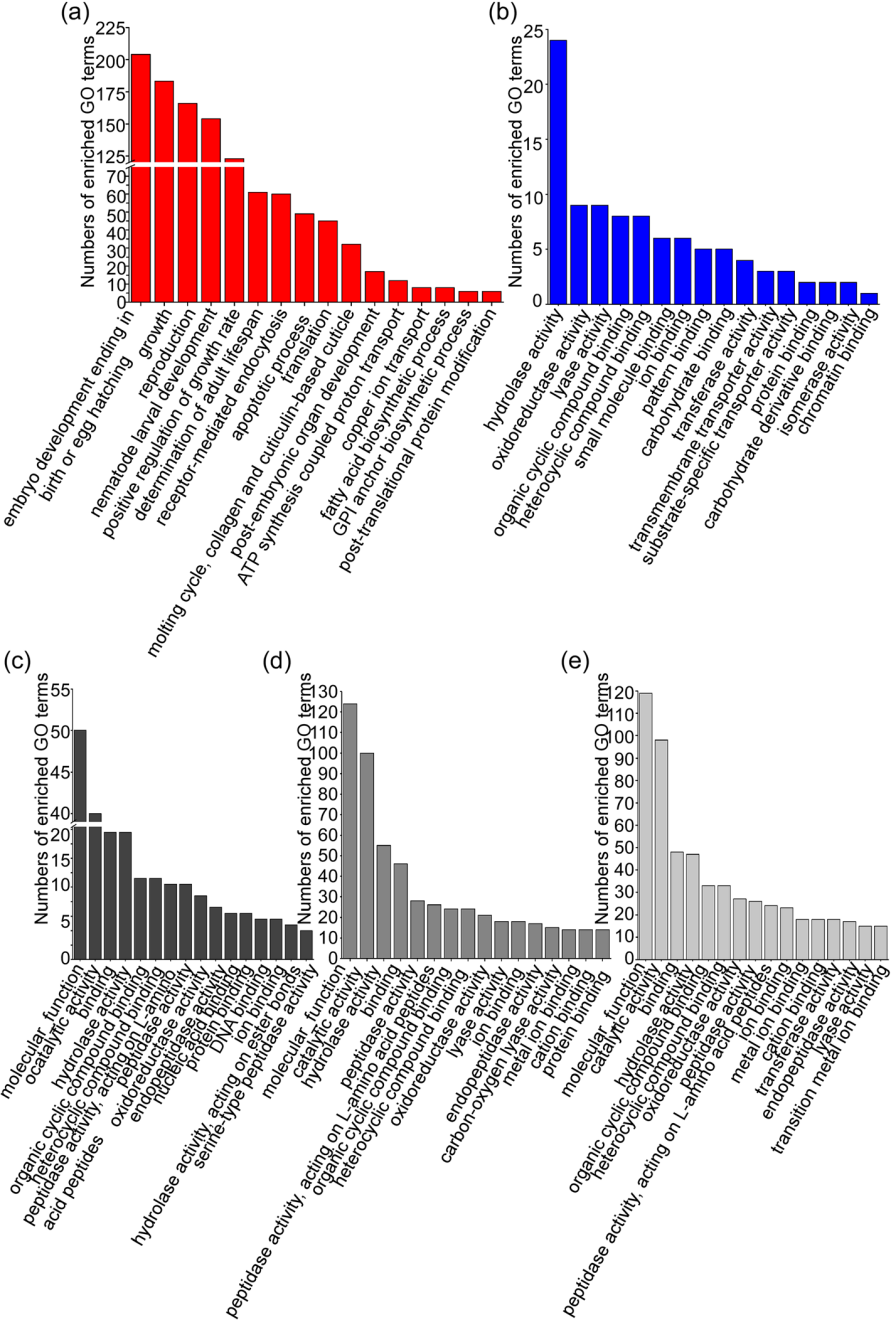
Top 16 Gene Ontology categories of upregulated genes in three *Bursaphelenchus xylophilus* transcriptomes using the standard method. **a** Representative GO terms in the *B. xylophilu*s transcriptomes at 2.5 h postinfection. **b** Representative GO terms in the *B. xylophilu*s transcriptomes at 6 and 15 d postinfection. **c**-**e** Representative GO terms in the *B. xylophilu*s transcriptomes at 6, 12 and 24 h postinfection

Transcriptome annotation provides insight into the structural, functional and biological processes in which genes are involved [20]. To determine the functional annotation of upregulated *B. xylophilus* genes, we used the GO database. The GO terms of the upregulated genes from the three *B. xylophilus* transcriptomes were compared, and the transcriptomes ranked in the top 16 terms in the molecular function category are shown in Fig. 1. The results showed that the major functions of the upregulated genes involved were biological processes of *B. xylophilus*, such as embryo development, positive growth rates and reproduction. This result indicated that the genes related to the growth and development of *B. xylophilus* played a role in the initial stage of the plant-host interaction (2.5 h postinfection) (Fig. 1a). The most highly represented GO terms in this set of 200 upregulated genes of *B. xylophilus* in the molecular function category were hydrolase, oxidoreductase and lyase activity in the middle and later stages of infection (6 and 15 d postinfection) (Fig. 1b). In our experimental data, the most highly represented GO terms of the 247 upregulated genes in the molecular function category were molecular function, catalytic activity, hydrolase activity and binding at the early stage of infection regardless of the time points (6, 12 and 24 h postinfection) (Fig. 1c-e). At the same time, the same GO terms were highly represented in the latter two transcriptomes in the molecular function category, such as hydrolase, oxidoreductase activity, lyase activity and ion binding. These findings indicated that *B. xylophilus* uses some similar responses to survive in pines.

### Identification of candidate effectors in three *B. xylophilu*s transcriptomes

In our previous experimental data, 69 out of the 247 genes were denoted as candidate effectors [15]. According to the accepted screening criterion of candidate effectors, we screened 62 candidate effectors in the *B. xylophilu*s transcriptome at 2.5 h postinfection. Moreover, 46 candidate effectors were identified by Espada et al. [14]. The list of candidate effectors from the three transcriptomes is shown in Table S3. To compare the candidate effectors from different *B. xylophilu*s transcriptomes, these candidate effectors were matched with each other by local BLAST search. The results showed that seven candidate effectors were found in both our study and *B. xylophilu*s transcriptome at 2.5 h postinfection, and one candidate effector was found in all three *B. xylophilu*s transcriptomes (Table 1). These results showed that some effectors of *B. xylophilus* played a role in the early infection stage and that some effectors played a role in the middle and later infection stages. That is, similar to other pathogens, *B. xylophilu*s secretes different effectors at different infection stages. Nevertheless, there are also some effectors that function throughout the whole infection process.

**Table 1:** List of common candidate effectors in three transcriptomes of *Bursaphelenchus xylophilus*

No.	Gene ID	Signal peptide (aa)	Length of amino acid sequence (aa)	Blast swiss prot	Note
1	BXY_0583800 (i.e. BxSapB1)	16	145	SapB domain	Common candidate effectors appeared in *B. xylophilu*s transcriptomes at 2.5 h postinfection and 6, 12 and 24 h postinfection
2	BXY_0495300 (i.e. BxSapB3)	16	167	SapB domain	
3	BXY_168500 (i.e. Bx-FAR-1)	19	166	FAR-1 domain	
4	BXY_0588800 (namely Bx-C1)	16	205	None	
5	BXY_1074200 (namely Bx-C2)	19	452	Epoxide hydrolase 1	
6	BXY_1014800 (namely Bx-C3)	17	734	Neprilysin-1	
7	BXY_1076300 (namely BxSCD5)	16	156	None	
8	BXY_1014700 /BUX.s01661.67 (namely Bx-C4)	17	731	Neprilysin-1	Common candidate effector appeared in the three *B. xylophilus* transcriptomes

A previous study has characterized a *B. xylophilus* secretome during *P. thunbergii* infection [21]. To determine the number of our predicted candidate effectors also identified as secreted proteins in this study, all the candidate effectors from three transcriptomes were matched to the secretome by local BLAST search. The results showed that 59, 29 and 24 candidate effectors from the *B. xylophilu*s transcriptome at 2.5 h postinfection, 6, 12 and 24 h postinfection and 6 d and 15 d postinfection were identified in the secretome of *B. xylophilus*, respectively (Table S4). The results indicated some cross-validation between these studies.

### Functional annotation of candidate effectors

**Fig. 2:**
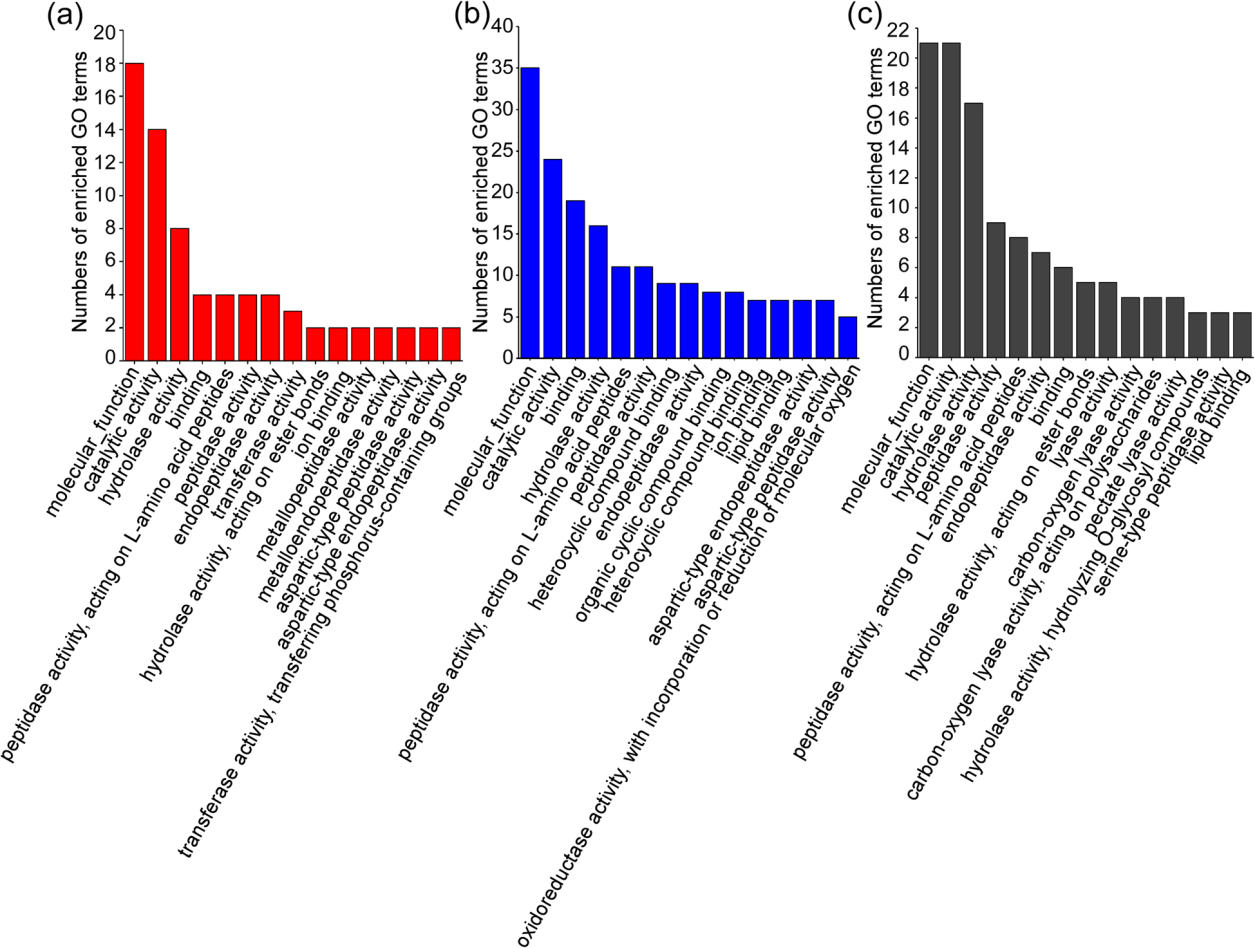
Top 16 Gene Ontology categories of candidate effectors in three *Bursaphelenchus xylophilus* transcriptomes using the standard method. **a** Representative GO terms in the *B. xylophilu*s transcriptomes at 2.5 h postinfection. **b** Representative GO terms in the *B. xylophilu*s transcriptomes at 6, 12 and 24 h postinfection. **c** Representative GO terms in the *B. xylophilu*s transcriptomes at 6 and 15 d postinfection

To determine the functional annotation of *B. xylophilus* candidate effectors screened from different infection stages, we also used the GO database. Because the GO annotation of the candidate effectors for 6 and 15 d of *B. xylophilus* infection in pine has not been published, we matched 46 candidate effectors to our transcriptome data by local BLAST search and found the corresponding gene ID numbers. Then, we searched for the GO annotation of these genes in our transcriptome GO annotation information. The GO terms ranked in the top 15 in the molecular function are shown in Fig. 2. The results showed that the most highly represented GO terms of candidate effectors from three transcriptomes in the molecular function category were all molecular function, catalytic activity and hydrolase activity and that 8 out of 15 GO terms were the same between the transcriptomes of pines infected with PWN for 2.5 h and 6, 12 and 24 h (Fig. 2a, c). Ten out of 15 GO terms were the same between the transcriptomes of pines infected with PWN for 2.5 h and 6 d and 15 d (Fig. 2a-b), and 7 out of 15 GO terms were the same between the transcriptomes of pines infected with PWN for 6, 12 and 24 h and 6 and 15 d (Fig. 2b-c). This result indicated that candidate effectors from the three transcriptome groups played similar roles in the infection stage, and their hydrolase activity and catalytic activity played an important role in degrading the plant cell wall and removing ROS by catalysis, which helped *B. xylophilus* to successfully infect pine.

### Categories of candidate effectors from three *B. xylophilu*s transcriptomes

**Fig. 3:**
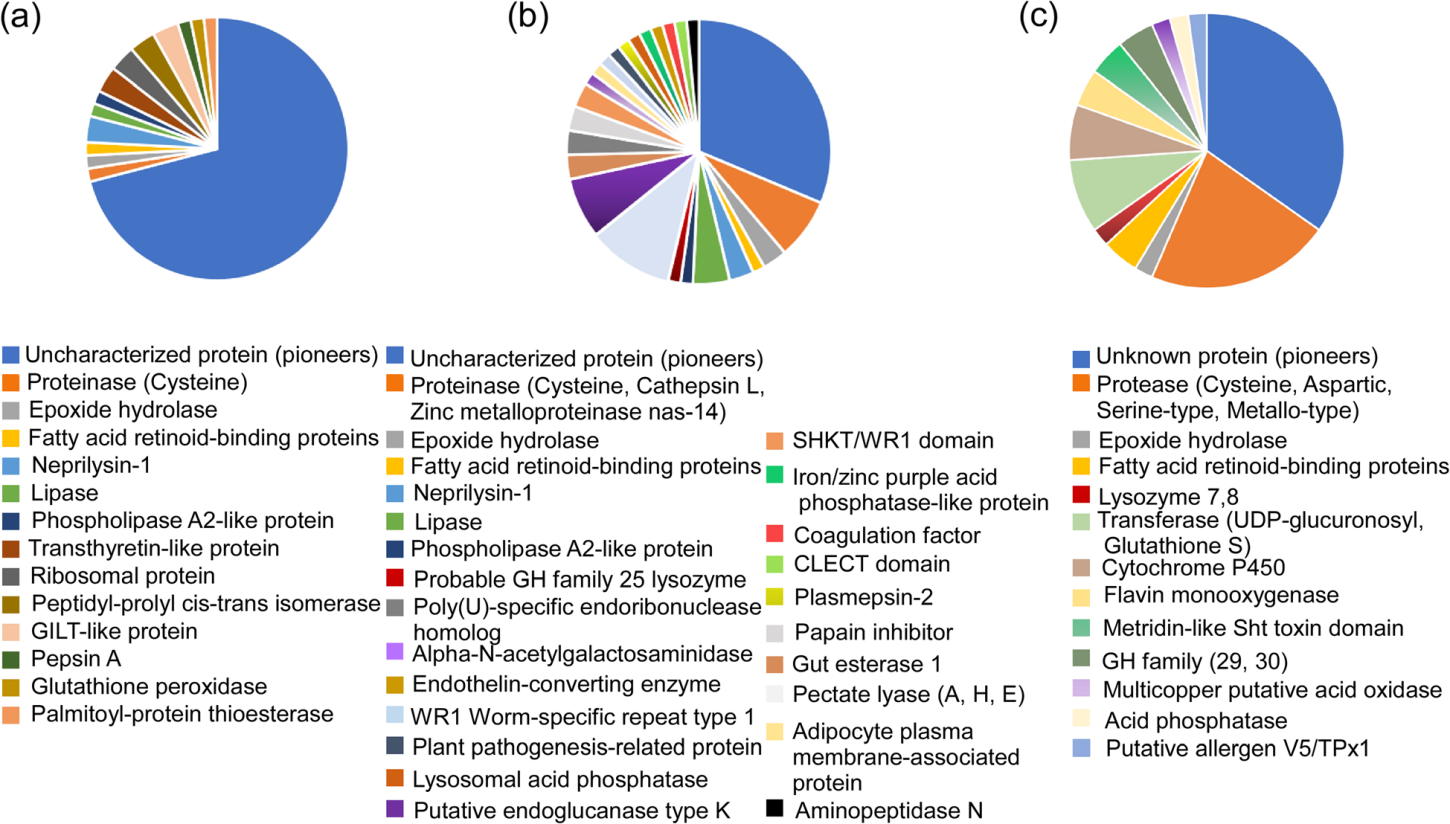
Annotation information of candidate effectors in the SWISS-Prot database. **a** Annotation information of candidate effectors in *Bursaphelenchus xylophilus* transcriptomes at 2.5 h postinfection. **b** Annotation information of candidate effectors in the *B. xylophilu*s transcriptomes at 6, 12 and 24 h postinfection. **c** Annotation information of candidate effectors in the *B. xylophilu*s transcriptomes at 6 and 15 d postinfection

The candidate effectors were searched in the SWISS-Prot database by using BLASTP. The categories of candidate effectors from the three transcriptomes are shown in Fig. 3. Forty-four out of 62 candidate effectors from the *B. xylophilu*s transcriptome at 2.5 h postinfection were uncharacterized proteins; thus, they were denoted as pioneers. Moreover, the other candidate effectors included several classes of proteases (pepsin A and cysteine proteinase), transthyretin-like protein, ribosomal protein and lipase as well as several enzymes involved in the detoxification of xenobiotic compounds, such as epoxide hydrolase and glutathione peroxidase (Fig. 3a). Twenty-one out of 69 candidate effectors from the *B. xylophilus* transcriptome at 6, 12 and 24 h postinfection were pioneers. The other candidate effectors included proteases (such as cysteine proteinase B, cathepsin L and zinc metalloproteinase nas-14), carbohydrate active enzymes (CAZymes; such as pectate lyase A, pectate lyase H, pectate lyase E, and putative endoglucanase type K), lipase, probable GH family 25 lysozyme and detoxification-related proteins, including lysosomal acid phosphatase, iron/zinc purple acid phosphatase-like protein and epoxide hydrolase (Fig. 3b). Sixteen out of 46 candidate effectors from the *B. xylophilus* transcriptome at 6 and 15 d postinfection were pioneers. The other candidate effectors also included several classes of proteases (aspartic protease A1, cysteine proteases C1A and serine-type protease), fatty acid transport proteins, putative V5/TPx1 allergen, a lysozyme and several detoxification-related proteins, such as UDP-glucuronosyl transferase, multicopper putative acid oxidase, glutathione S-transferase, cytochrome P450, acid phosphatase and epoxide hydrolase (Fig. 3c).

### Comparison of the KEGG pathway distribution for candidate effectors from the three *B. xylophilu*s transcriptomes

The candidate effectors from three *B. xylophilus* transcriptomes were analyzed by BLAST using the KEGG pathway database with KOBAS software. The results showed that most candidate effectors were not assigned to known KEGG pathways (Table 2). Only one candidate effector (BXY_1074200) from the *B. xylophilu*s transcriptomes at 2.5 h postinfection was assigned to the metabolism of xenobiotics by the cytochrome P450 pathway. At the same time, only one candidate effector (BXY_0298700) from the *B. xylophilu*s transcriptomes at 6 and 15 d postinfection was assigned to the drug metabolism-cytochrome P450 pathway. Seven candidate effectors from the *B. xylophilu*s transcriptomes at 6, 12 and 24 h postinfection were assigned to four pathways as follows: drug metabolism-other enzymes, metabolic pathways, lysosomes and metabolism of xenobiotics by cytochrome P450 pathways. This result indicated that the cytochrome P450 pathway is important to *B. xylophilu*s during the entire infection stage.

**Table 2:** The KEGG pathway list of known enriched candidate effectors of *Bursaphelenchus xylophilus*

#Term	Gene ID	Database	ID	Note
Metabolism of xenobiotics by cytochrome P450	BXY_1074200	KEGG PATHWAY	cel00980	appeared in *B. xylophilu*s transcriptomes at 2.5 h postinfection
Drug metabolism - other enzymes	BXY_0867200	KEGG PATHWAY	cel00983	appeared in *B. xylophilu*s transcriptomes at 6, 12 and 24 h postinfection
Metabolic pathways	BXY_0407400		cel01100	
	BXY_0867200			
Lysosome	BXY_0832500		cel04142	
	BXY_0842200			
Metabolism of xenobiotics by cytochrome P450	BXY_0298000		cel00980	
	BXY_1074200			
Drug metabolism - cytochrome P450	BXY_0298700	KEGG PATHWAY	cel00982	appeared in *B. xylophilu*s transcriptomes at 6 and 15 d

### Eight common candidate effectors are highly upregulated at early infection stages

**Fig. 4:**
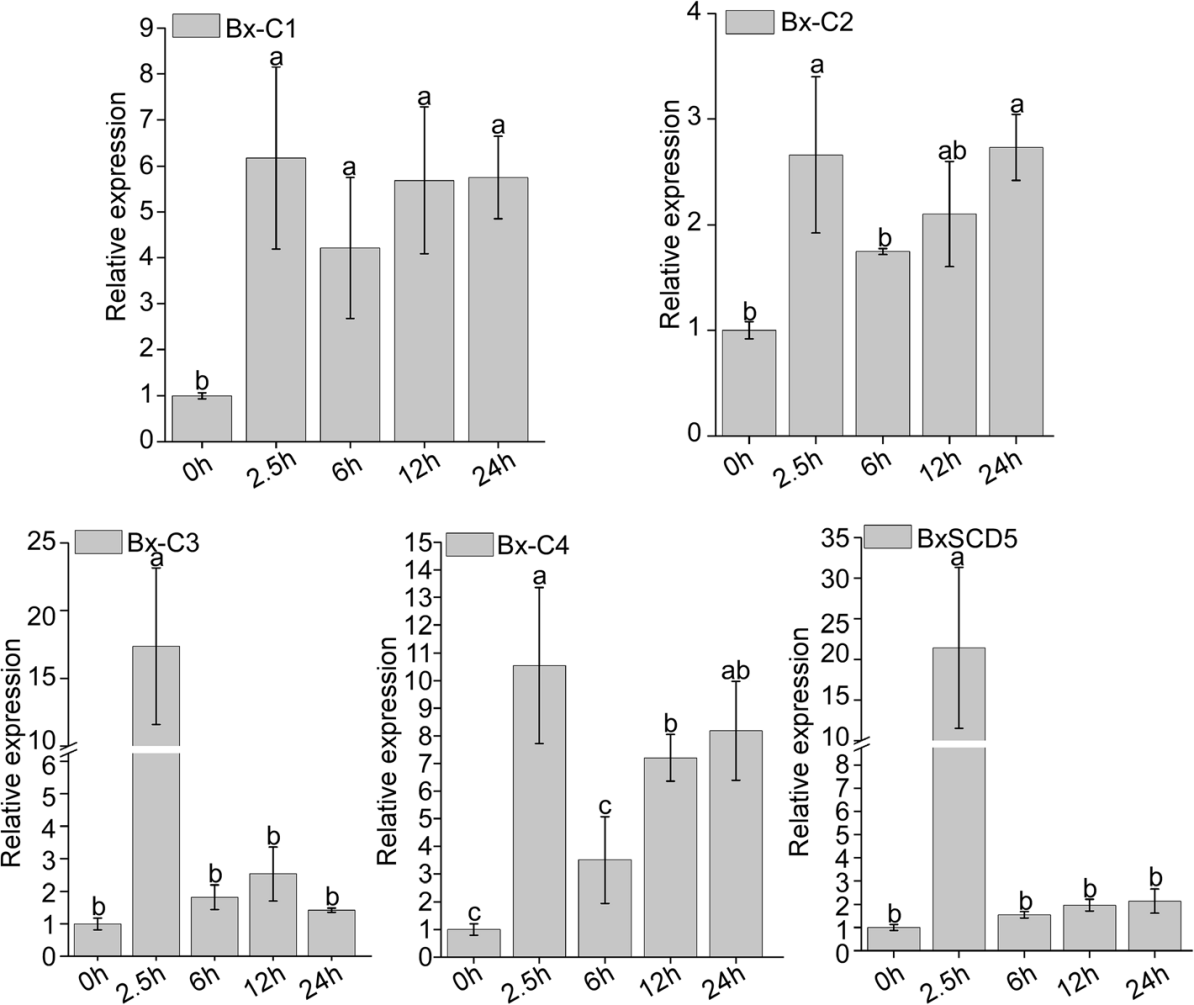
The expression pattern of the five common candidate effectors from the three transcriptome datasets for *Bursaphelenchus xylophilus* at the early infection stages by quantitative real-time polymerase chain reaction (qRT-PCR) analysis. Relative expression of the five candidate effectors at the mycetophagous stage (0 h) and at the early stages of infection (2.5, 6, 12 and 24 h). Data represent the means, and the error bars represent ± SD from three biological replicates. Different letters on top of the bars indicate statistically significant differences (*p* < 0.05, t test) as measured by Duncan’s multiple range test

According to the above transcriptome data for *B. xylophilus*, 8 common candidate effectors (including three known effectors, namely, BxSapB1, BxSapB3 and Bx-FAR-1) were upregulated at the infection stages. In addition to BxSapB1, BxSapB3 and Bx-FAR-1, the other five common candidate effectors were named *Bx-C1*, *Bx-C2*, *Bx-C3*, *Bx-C4* and *BxSCD5*. To further confirm this finding, qRT-PCR was employed to obtain their expression profiles at the early stages of infection. The results showed that the five common candidate effectors were upregulated at early infection stages compared to the mycophagous stage. The relative expression levels of *Bx-C1*, *Bx-C3*, *Bx-C4* and *BxSCD5* were highest at 2.5 h compared to other infection times, and the relative expression of *Bx-C2* was highest at 24 h (Fig. 4). These results suggested that these common candidate effects play important roles in the stages of *B. xylophilus* infection.

### BxSCD5 effectively inhibits PAMP PsXEG1-triggered cell death

**Fig. 5:**
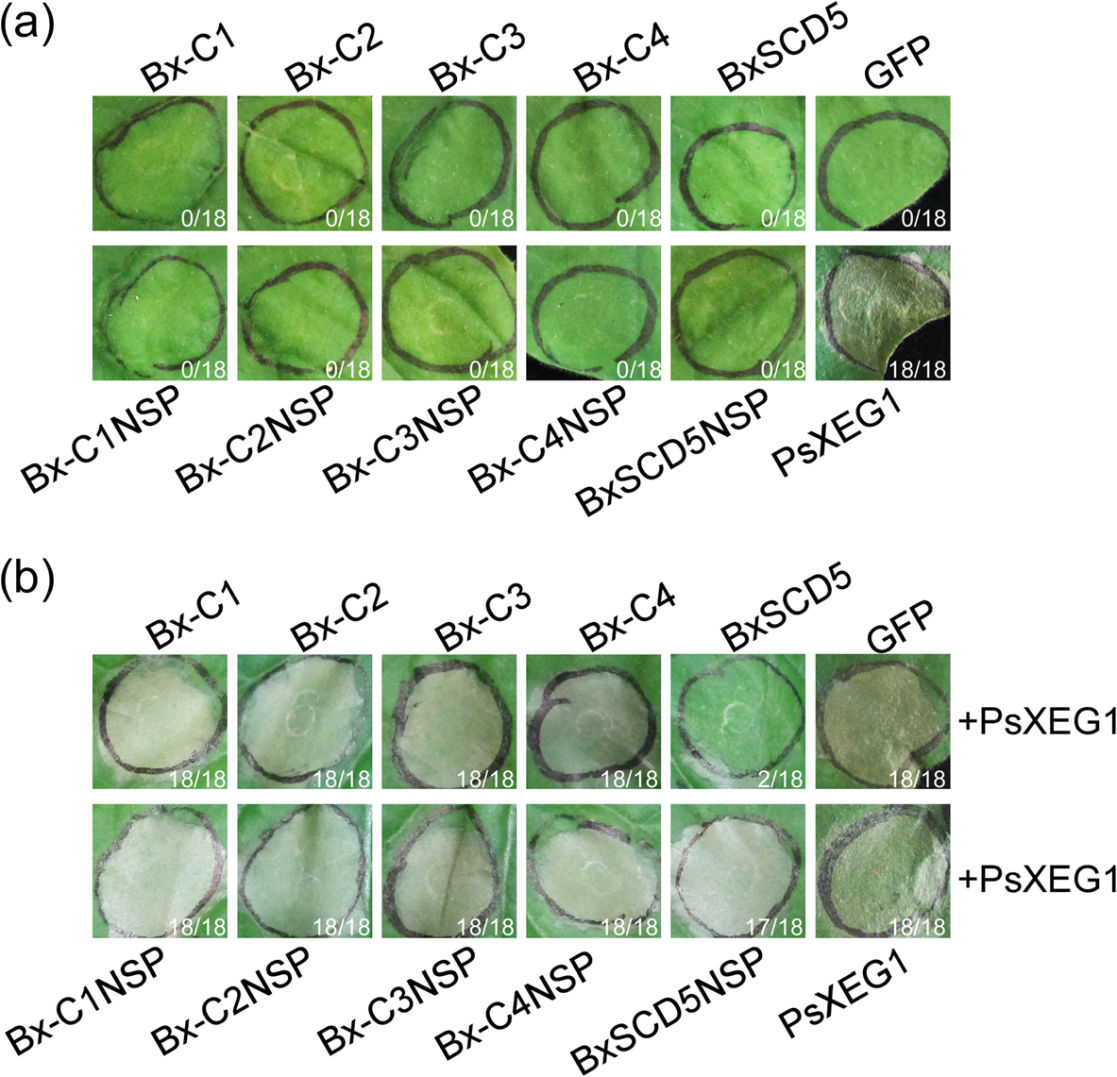
The BxSCD5 candidate effector of *Bursaphelenchus xylophilus* suppresses PAMP PsXEG1-triggered cell death in *Nicotiana benthamiana*. **a** Representative *N. benthamiana* leaves at 7 days after inoculation with *Agrobacterium* sp. strain GV3101 carrying five common candidate effectors [with and without native signal peptide (SP)] of *B. xylophilus* in the pGR107 vector. Ratios in the picture indicate the proportion of infiltrated sites that developed the cell death phenotype. The assay was repeated at least three times. In each assay, three different plants with three inoculated leaves were used, yielding similar results. **b** Functional detection of five common candidate effectors (with and without native SP) of *B. xylophilus* to inhibit PAMP PsXEG1-triggered cell death. Ratios in the picture indicate the proportion of infiltrated sites that developed the cell death phenotype. The infiltration assay was performed three times, and in each assay, three different plants with three inoculated leaves were used, yielding similar results

A previous study has shown that some *Phytophthora* effectors cause cell death in host and/or nonhost plants, such as the *P. sojae* RXLR effector, Avh241 [22]. To test whether the 5 common candidate effectors of *B. xylophilus* induce cell death, they were expressed in *N. benthamiana* leaves using agroinfiltration. The results showed that the proportion of infiltrated sites that developed the cell death phenotype after injection of the 5 candidate effectors was almost zero, indicating that they do not trigger cell death in *N. benthamiana* (Fig. 5a).

PAMPs trigger strong defense responses in various plants, and many *P. sojae* effectors suppress immune responses, including PAMP-triggered cell death [19]. To determine whether the 5 common candidate effectors (except BxSapB1, BxSapB3 and Bx-FAR-1) of *B. xylophilus* suppress *P. sojae* PAMP PsXEG1-triggered cell death, we expressed PsXEG1 in *N. benthamiana* leaves 16 h after expressing the 5 common candidate effectors or GFP (negative control) using agroinfiltration. The proportion of infiltrated sites that developed the cell death phenotype after injection of BxSCD5NSP (without SP) and the other 4 candidate effectors followed by injection of PsXEG1 was almost 100%, except for BxSCD5 (with SP). These results showed that only BxSCD5 suppressed PsXEG1-triggered cell death (Fig. 5b), indicating that BxSCD5 suppresses PsXEG1-triggered cell death in *N. benthamiana* when secreted into the apoplast.

## Discussion

It is generally accepted that the DEGs of pathogens, especially upregulated genes, are involved in the interaction between pathogens and hosts during the infection stage. In this study, the raw reads, clean reads and upregulated genes obtained by the three transcriptome groups were different, and the annotation information for the upregulated genes showed that the gene functions in *B. xylophilus* infection were different in different periods. In the A group (2.5 h postinfection), the upregulated genes were mainly involved in the growth and development of *B. xylophilus*, which may be due to adjustments made by *B. xylophilus* to survive and better adapt to the new environment during the sudden change from the mycetophagous stage to the phytophagous stage. In the B group (6, 12 and 24 h postinfection), the major upregulated genes were involved in molecular function, catalytic activity, hydrolase activity and binding, and the number of upregulated genes involved in these GO terms was vast. In the C group (6 and 15 d postinfection), the upregulated genes were mostly involved in hydrolase, oxidoreductase and lyase activities. Interestingly, the number of upregulated genes involved in hydrolase, oxidoreductase and lyase activities in the B group was greater than that in the C group. This result indicated that it was more important for *B. xylophilus* to degrade the cell wall of the host by secreting hydrolase and lyase to promote parasitism at the early infection stage. Previous studies have demonstrated that some plant cell wall-degrading enzymes (CWDEs) of *B. xylophilus* have roles as pathogenicity determinants, such as glycoside hydrolase family 45 cellulases, pectate lyases and b-1,3-endoglucanases [23–25]. In this study, several pectate lyases and endoglucanases were identified as candidate effectors in the B group, supporting the viewpoint that these CWDEs are generally regarded as “effectors”. Moreover, an oxidative burst is one of the earliest defense responses to plant pathogen attack. The transient accumulation of ROS helps to defend the host from invading pathogens and also acts as a signaling molecule to trigger various other plant defense responses [26]. Some previous studies have shown that the capability of ROS scavenging in the host is pivotal to the high resistance of PWN and that enhancement of oxidative stress contributes to increased pathogenicity of *B. xylophilus* [27, 28]. The amount of ROS in the tree is critical to the survival of *B. xylophilus*. In addition, catalases (*Bxy-ctl-1* and *Bxy-ctl-2*) of high virulence *B. xylophilus* are crucial for nematode survival under prolonged exposure to in vitro oxidative stress [29]. Surprisingly, *Bxy-ctl-1* (BUX.s00579.159) was identified in the B group (i.e., BXY_1386500) with oxidoreductase activity, participating in the oxidation-reduction process. Moreover, the significant GO terms of oxidoreductase activity and oxidation-reduction process of virulent *B. xylophilus* strain were enriched statistically compared to the avirulent strain [30]. In our study, the significant GO term, oxidoreductase activity (GO:0016491), was found in both the B and C groups, and the DEG list of enriched statistically terms was mostly in the B group. Thus, we speculated that the upregulated genes functioning in catalytic and oxidoreductase activities in the B and C groups may play a key role in removing ROS by catalysis and oxidoreduction to help *B. xylophilus* survive in the host.

The genomic data of *B. xylophilus* indicate that this nematode has many specific genes [31]. A previous study has shown that effector repertoires of sedentary PPNs (e.g., cyst and root-knot nematodes) contain hundreds of proteins implicated in the establishment of a successful plant-pathogen interaction [7]. In this study, although the number of upregulated genes in the three transcriptomes was different, the number of candidate effectors was similar (62, 69 and 46), indicating that the number of effectors of *B. xylophilus* may be less than that of sedentary PPNs. Although the life cycle of *B. xylophilus* is different from that of other PPNs, homologous genes in different PPNs often play similar functions. Several candidate effectors screened in the present study have also been studied in other PPNs. For example, glutathione S transferase (GST) has been demonstrated to play an important role in root-knot nematodes [32], and GST was found in the C group in the present study. Fatty acid retinoid-binding (FAR) proteins have also been studied in several PPNs (such as *Radopholus similis*, *Globodera pallida* and *P. penetran*), indicating that FAR proteins are necessary for the entire nematode life cycle [33–35]. In the present study, *B. xylophilus* FAR proteins were identified in the three groups. Moreover, Bx-FAR-1 has been characterized as a key effector by Li [18]. The VAPs of the *G. rostochiensis* potato cyst nematode are required for the suppression of host immunity [36]. In the present study, one candidate effector similar to VAPs was identified in the C group.

CAZymes (such as cellulase, pectate lyase, calreticulin and expansin) are important for breaking down the polysaccharides of plant cell walls to establish infection [8]. In the present study, some CAZymes (putative pectate lyase A, H and E as well as endoglucanase type K) were included in the candidate effectors listed in the B group, which may play roles in invasion, extension and degradation of host tissues. Fatty acid-binding proteins, retinol-binding proteins and peroxiredoxin have been reported to protect nematodes from antipathogen compounds in host defenses [37]. In the present study, fatty acid- and retinol-binding proteins were identified in all groups. Some effectors, such as annexin, venom allergen-like protein, transthyretin-like protein and ubiquitin extension proteins, target plant signaling pathways and suppress host defenses [37]. Here, the putative allergen, V5/TPx1, and transthyretin-like protein were identified in the C and A groups, respectively. A previous study has shown that some effectors are required for feeding efficiency, such as cathepsin. Proteolytic enzymes can be divided into four main groups as follows: cysteine, serine, aspartyl and metalloproteinases. Among them, cysteine proteinases are the most extensively studied [38]. In our study, cysteine proteinases, including cathepsin L, were found in all three transcriptome groups. These results indicated that proteolytic enzymes, especially cysteine proteinases, of *B. xylophilus* play a key role in nutritional intake to promote parasitism. Some studies have also shown that cysteine proteinases play important roles in embryogenesis, development, infection, parasitism, pathogenesis and immune evasion in nematodes and many other animal parasites [39–41]. Moreover, a cathepsin L-like cysteine proteinase of *B. xylophilus* has been demonstrated to influence its development and pathogenicity [42]. Several lipases of pathogenic bacteria and fungi have been identified as virulence factors [43]. Here, several other digestive enzymes, such as serine-type protease, pepsin A and aspartic protease A1, were also identified in the three groups, and lipase was present in both the A and B groups. Many putative effectors have unknown functions and are called pioneers. In the present study, many pioneers were identified in three transcriptome groups, and most of them successfully matched the secretome of *B. xylophilus* [21]. The functional study of these pioneers will be an important step to investigate the pathogenesis of *B. xylophilus*.

In addition to the above functions, we found that some effectors were related to the detoxification of xenobiotic compounds. For example, epoxide hydrolase and glutathione peroxidase were identified in the A group. Epoxide hydrolases have been characterized as virulence factors in previous studies [44, 45]. Unlike epoxide hydrolase, lysosomal acid phosphatase and iron/zinc purple acid phosphatase-like protein were included in the B group. UDP-glucuronosyl transferase, multicopper putative acid oxidase, GST, cytochrome P450 and acid phosphatase were categorized in the C group. This result suggested that both early and late infection stages of *B. xylophilus* require detoxification to overcome host resistance, but some detoxification substances secreted by *B. xylophilus* are different. Moreover, several candidate effectors in the A group, such as ribosomal proteins, were suggested to participate in RNA transcription and translation. Additionally, 17 genes encoding ribosomal proteins (RPs) have been found to be significantly altered when *Meloidogyne incognita* infects *Solanum lycopersicum* roots [46]. These results indicated that ribosomal proteins play an important role in the interaction of phytoparasitic nematodes and their hosts.

In the process of interaction between pathogens and hosts, the secretion of PAMPs and effectors by pathogens can trigger plant defense responses, including PAMP-triggered immunity (PTI) and effector-triggered immunity (ETI). However, under the strong pressure of natural selection, pathogens will continue to produce new effectors to inhibit the PTI and ETI of the host and help pathogens escape host recognition [47]. At the same time, synergistic cooperation between effectors can promote the successful infection of pathogens [3]. A previous study has shown that the expression of some PR genes of pine is induced by infection with virulent isolates of *B. xylophilus* [48], indicating that *B. xylophilus* might secrete some PAMPs or molecular patterns, such as BxCDP1 [17], to induce the defense response of pines. In our study, we transiently expressed the five common candidate effectors (except the three known effectors, BxSapB1, BxSapB3 and Bx-FAR-1) in the *N. benthamiana* model plant for functional analysis. Among the five candidate effectors, the BxSCD5 candidate effector effectively suppressed PAMP PsXEG1-triggered cell death in *N. benthamiana*. Thus, we speculated that although the defense response of the host is induced after infection with *B. xylophilus*, there are also some effectors that inhibit host defense to some extent to help nematodes successfully infect, such as BxSCD5. Effectors of many pathogens have been identified using this strategy [49–52]. Thus, it is also feasible to identify effectors of *B. xylophilus* in the *N. benthamiana* nonhost plant. Certainly, the role effectors play in the pathogen-host interaction process still needs to be studied in their own host. Thus, the function of BxSCD5 in host pines will be studied in the future.

In the present study, common effectors (7 candidate effectors were in both the A and B groups, and 1 candidate effector was in all three groups) were identified in the three *B. xylophilus* transcriptomes. There are several possibilities why only a few factors were identified. First, similar to other pathogens, *B. xylophilus* secretes different effectors at different infection stages to cope with various survival threats, resulting in a few common effectors in three different infection stages. Second, only the top 200 upregulated genes in the parasitic life stage of the nematode were selected to predict candidate effectors in the C group (6 and 15 d postinfection) [14]; thus, the candidate effectors were screened from the top 200 upregulated genes, not from all of the upregulated genes, which might result in the loss of some effectors. Third, different *B. xylophilus* strains, pine species, tree age, inoculation environment and sequencing depth of the three transcriptomes used might lead to different DEGs and candidate effectors. Nevertheless, the BxSapB1, BxSapB3, Bx-FAR-1 and BxSCD5 key effectors were still screened from the three different *B. xylophilus* transcriptomes and demonstrated to play an important role in promoting nematode infection. Thus, despite its shortcomings, the relative comparative transcriptomic analysis used in the present study is feasible to some degree. To fully understand the mechanism of *B. xylophilus* infection, we will also study the function of noncommon candidate effectors from the three transcriptomes in the future.

## Conclusions

The aim of the present study was to explore infection and survival strategies by comparative transcriptomic analysis of upregulated genes and candidate effectors of *B. xylophilus* in different infection stages. *B. xylophilus* secretes various candidate effectors in different infection stages with some of them continuing to function throughout all infection stages. Candidate effectors help *B. xylophilus* infect and survive successfully in hosts in different ways (such as breaking down host cell walls, suppressing host defenses, promoting feeding efficiency, detoxification and playing virulence functions). We provided continued evidence for the presence of ‘common’ PPN effectors and identified some novel effectors involved in the infection process of *B. xylophilus*. These results provide many resources for studying the pathogenesis of *B. xylophilus* from the perspective of effectors. Moreover, the identification and characterization of the plant targets of these effectors should also be undertaken to understand how these effectors hijack many aspects of plant cell morphology and physiology, including the immune system.

## Methods

### Biological material

In this study, the highly virulent *B. xylophilus* strain AMA3 was used, which was from Anhui Province, China [53]. The culture methods of *B. xylophilus* were similar to those previously reported [54]. The culture and isolation of nematodes were performed according to our previous study [15].

### PWN inoculation trials

*Pinus thunbergii* seedlings (3 years old) obtained from the experimental field of Nanjing Forestry University (Jurong Yaolingkou Forest Farm, Jiangsu, China) were used for inoculation of the PWN strain AMA3. The number of nematodes and time of inoculation have been described in our previous study [15]. Briefly, a suspension of approximately 10,000 mixed life stage nematodes was collected from PDA as a mycetophagous control (B-0 h). The same number of nematodes (AMA3) was inoculated into the pine stems for 6 h (B-6 h), 12 h (B-12 h) and 24 h (B-24 h). The nematodes were collected and then frozen for further RNA isolation according to our previous method [15]. Each treatment had three biological replicates.

### Sample preparation and Illumina sequencing

Nematode (AMA3) RNA was extracted from the B-0 h, B-6 h, B-12 h and B-24 h samples. Then, mRNA degradation and contamination were monitored on 1% agarose gels. Then, the RNA purity, RNA concentration, RNA integrity, cDNA library preparations and sequencing were separately determined according to our previous study [15]. The RNA-seq data for this study are submitted on the NCBI under accession number PRJNA397001.

### Differential gene expression analysis

HTSeq v0.6.1 and DESeq were used to count the read numbers mapped to each gene and standardize the read count of each gene, respectively. Differential expression analysis of four treatments was performed using the DESeq R package (1.18.0), which has been previously demonstrated to be better than FPKM or RPKM [55]. Finally, genes (with a *P*-value < 0.05 and log_2_ (fold change) > 1) were assigned as differentially expressed among four treatments. The definition of DEGs were described in our previous study [15].

### Bursaphelenchus xylophilus transcriptomic datasets

The two previously reported *B. xylophilus* transcriptomic datasets from different stages of infection (2.5 h postinfection and 6 d and 15 d postinfection) [13, 14] were collected for subsequent relative comparative transcriptomic analysis with our transcriptomic experimental data. Among them, one experimental design used two postinoculation events (2.5 h postinfection in August and September) and one control (mycetophagous stage) with two biological replicates per condition, which were denoted as A-2.5 h-8, A-2.5 h-9 and A-0 h. The other dataset also used two postinoculation events (6 and 15 d postinfection) and one control (mycetophagous stage), which were denoted as C-6 d, C-15 d and C-0 d. The two postinoculation events of the C group had three biological replicates, and the control had two biological replicates.

### GO enrichment analysis of differentially expressed genes

The GO enrichment analysis of *B. xylophilus* DEGs from the above three transcriptomic datasets was implemented by the GOseq R package, in which gene length bias was corrected [56]. The GO terms were divided into biological processes, cellular components and molecular functions for functional categorization. GO terms with corrected *P* values (i.e., false discovery rate, FDR) less than 0.05 were considered significantly enriched.

### Candidate effectors analysis

We screened potential effector proteins according to our previous study (the presence of an N-terminal signal peptide and the absence of a transmembrane domain) [15]. The signal peptide and the transmembrane domain were predicted by SignalP 4.1 (http://www.cbs.dtu.dk/services/SignalP/) [57] and TMHMM 2.0 (http://www.cbs.dtu.dk/services/TMHMM/) [58], respectively. All candidate effectors were predicted functions according to our previous method [15].

### KEGG enrichment analysis of candidate effectors of *B. xylophilus*

KEGG is a database resource for understanding the high-level functions and utilities of biological systems (http://www.genome.jp/kegg/) [59]. KOBAS software was used to determine the statistical enrichment of *B. xylophilus* candidate effectors from the above three transcriptomic datasets in KEGG pathways. Furthermore, to improve the annotation, a BLAST search was performed against the SWISS-Prot database.

### Local BLAST analysis

The amino acid sequences of candidate effectors from the above three transcriptomic datasets were matched to the secretome of *B. xylophilus* by a local BLAST search [21]. The BLAST search was performed with an e-value = 1e-10, and the proteins with percentage similarity > 80% were selected.

### qRT-PCR assays

Referring to a previously used method [17], approximately 10,000 AMA3 nematodes were inoculated into 3-year-old *P. thunbergii* seedlings, and the nematodes were then collected at 2.5, 6, 12 and 24 h after inoculation. RNA of AMA3 nematodes was extracted and reverse transcribed into cDNA. qRT-PCR assays were performed according to our previous method [15]. The expression levels of the five common candidate effectors (except the three known effectors, BxSapB1, BxSapB3 and Bx-FAR-1) from the above three *B. xylophilus* transcriptomes at the early stage of host infection (2.5, 6, 12 and 24 h) were measured. Actin of *B. xylophilus* (GenBank EU100952) was used as a constitutively expressed endogenous control gene [48]. All assays were performed three times. Primer sequences are provided in Table S2.

### Plasmid construction

Referring to the previously used method [17], five common candidate effectors from the above three *B. xylophilus* transcriptomes were cloned with and without its native signal peptide (SP) from *B. xylophilus* cDNA using the specific primers listed in Table S2. Subsequently, purified PCR products were ligated into the pGR107 vector (pGR107–3*HA) using the CloneExpress II One Step Cloning Kit (Vazyme, Nanjing, China) after confirmation by sequencing.

### Transient expression assays in *N. benthamiana*

The transformation method of the constructed plasmids, culture conditions of the *Agrobacterium tumefaciens* GV3101 strain and agroinfiltration assays were the same as those in our previous study [17]. One experimental design directly infiltrated *A. tumefaciens* suspensions harboring five candidate effectors (with and without its native SP) into the leaves of *N. benthamiana* using a needleless syringe. Another experimental design infiltrated *A. tumefaciens* suspensions harboring five candidate effectors (with and without its native SP) 16 h prior to those infiltrated with *Phytophthora sojae* pathogen-associated molecular pattern (PAMP) PsXEG1. Green fluorescent protein (GFP) was used as the negative control. The phenotypes of *N. benthamiana* leaves were recorded at 5–7 days after infiltration. Each experiment was repeated three times, and each experiment consisted of three plants with three inoculated leaves. Finally, we counted the proportion of infiltrated sites that developed the cell death phenotype to screen the effectors that triggered cell death or inhibited PsXEG1-triggered cell death.

## Supplementary Information

**Additional file 1: Table S1.** Comparison of summary data from three transcriptomes of *Bursaphelenchus xylophilus* at the phytophagous and mycetophagous stages. A: Transcriptome of *B. xylophilus* at 2.5 h postinfection. B: Transcriptome of *B. xylophilus* at 6, 12 and 24 h postinfection. C: Transcriptome of *B. xylophilus* at 6 and 15 d postinfection.

**Additional file 2: Table S2.** List of primers used in this study.

**Additional file 3: Table S3.** List of candidate effectors in three transcriptomes of pine wood nematodes *Bursaphelenchus xylophilus*.

**Additional file 4: Table S4.** List of candidate effectors in the *Bursaphelenchus xylophilus* secretome during *Pinus thunbergii* infection.

**Additional file 5: Fig. S1.** Pearson correlation between samples. bx-A: Mycophagous stage (0 h). bx-B: *Bursaphelenchus xylophilus* was inoculated into each of the pine stems for 6 h. bx-C: *B. xylophilus* was inoculated into each of the pine stems for 12 h. bx-D: *B. xylophilus* was inoculated into each of the pine stems for 24 h. Each treatment had three biological replicates.

**Additional file 6: Fig. S2.** Venn diagram of differentially expressed genes (DEGs) at the mycetophagous stage and three early phytophagous parasitic stages. A total of 867 DEGs were obtained.

**Additional file 7: Fig. S3.** Heatmap of the differentially expressed genes (DEGs) at the mycetophagous stage and three early phytophagous parasitic stages.

## Data Availability

The RNA-seq data in this study was available through the NCBI under accession number PRJNA397001. We have a permission to collect the plant samples we used in this study. All the data and materials that are required to reproduce these findings can be shared by contacting the corresponding author, Prof. Xiao-Qin Wu (xqwu@njfu.edu.cn).

## Abbreviations

PWN: Pine wood nematode
PWD: Pine wilt disease
*B. xylophilus*: *Bursaphelenchus xylophilus*
PPNs: Plant-parasitic nematodes
ESTs: Expressed sequence tags
RNA-Seq: RNA sequencing
*G. asiaticumrice*: Gymnosporangium asiaticumrice
*P. penetran*: *Pratylenchus penetrans*
*G. rostochiensis*: *Globodera rostochiensis*
DEGs: Differentially expressed genes
GO: Gene Ontology
KEGG: Kyoto Encyclopedia of Genes and Genomes
FDR: False discovery rate
PVX: Potato virus X
qRT-PCR: Quantitative real-time polymerase chain reaction
*N. benthamiana*: *Nicotiana benthamiana*
CAZymes: Carbohydrate active enzymes
CWDE: Cell wall-degrading enzymes
*P. sojae*: *Phytophthora sojae*
RPs: Ribosomal proteins
PDA: Potato dextrose agar
*A. tumefaciens*: Agrobacterium tumefaciens
ROS: Reactive oxygen species
