# Supplementary figures and images for: Comparative transcriptomic analysis of candidate effectors to explore the infection and survival strategy of Bursaphelenchus xylophilus during different interaction stages with pine trees
Source: BMC Plant Biol. 2021 May 19;21:224. doi: 10.1186/s12870-021-02993-9 (PMC8132355; doi:10.1186/s12870-021-02993-9)

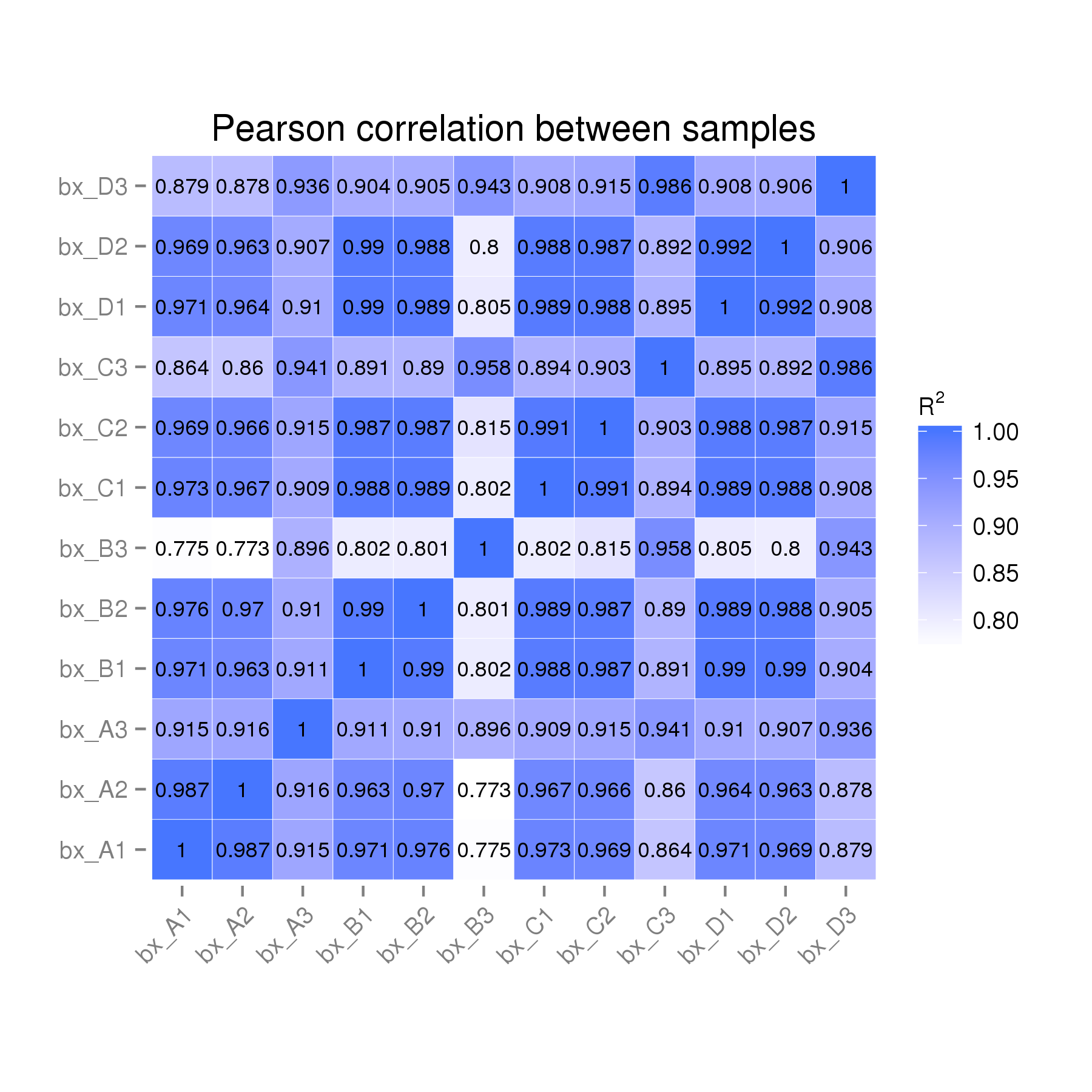

Supplement: Supplementary file 5 — Additional file 5: Fig. S1. Pearson correlation between samples. bx-A: Mycophagous stage (0 h). bx-B: Bursaphelenchus xylophilus was inoculated into each of the pine stems for 6 h. bx-C: B. xylophilus was inoculated into each of the pine stems for 12 h. bx-D: B. xylophilus was inoculated into each of the pine stems for 24 h. Each treatment had three biological replicates. [file 12870_2021_2993_MOESM5_ESM.tif]

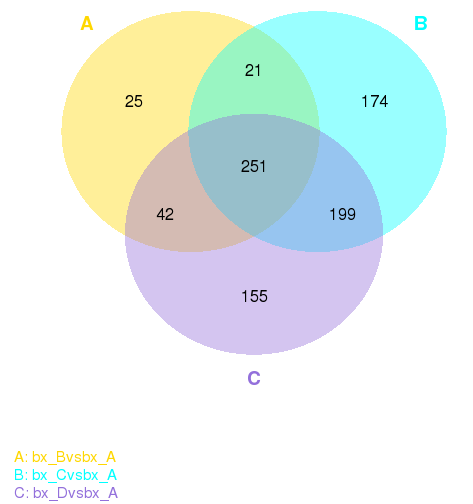

Supplement: Supplementary file 6 — Additional file 6: Fig. S2. Venn diagram of differentially expressed genes (DEGs) at the mycetophagous stage and three early phytophagous parasitic stages. A total of 867 DEGs were obtained. [file 12870_2021_2993_MOESM6_ESM.tif]

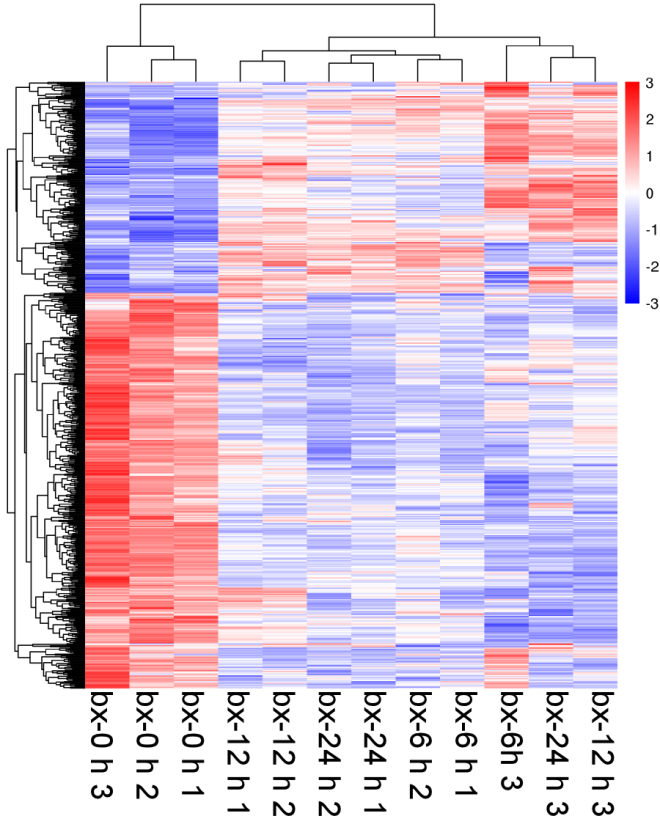

Supplement: Supplementary file 7 — Additional file 7: Fig. S3. Heatmap of the differentially expressed genes (DEGs) at the mycetophagous stage and three early phytophagous parasitic stages. [file 12870_2021_2993_MOESM7_ESM.tif]
